# Delineating the dispersal of Y-chromosome sub-haplogroup O2a2b-P164 among Austronesian-speaking populations

**DOI:** 10.1038/s41598-024-52293-z

**Published:** 2024-01-24

**Authors:** Javier Rodriguez Luis, Leire Palencia-Madrid, Göran Runfeldt, Ralph Garcia-Bertrand, Rene J. Herrera

**Affiliations:** 1 Department of Molecular Biology, Colorado College, 14 East Cache La Poudre Street, Colorado Springs, CO 80903-3294 USA; 2 Area de Antropología, Facultad de Biología, Universidad de Santiago de Compostela, Campus Sur s/n, 15782 Santiago de Compostela, Spain; 3 BIOMICs Research Group, Dpto. Z. y Biologia Celular A., Lascaray Research Centre, University of the Basque Country UPV/EHU, 01006 Vitoria-Gasteiz, Spain; 4 FamilyTreeDNA, Gene By Gene, Houston, TX 77008 USA

**Keywords:** Evolution, Phylogenetics

## Abstract

This article reports on an exploration of the Y-chromosome sub-haplogroup O2a2b-P164 in Austronesian-speaking populations. Moderate to high abundance of the P 164 mutation is seen in the West Pacific including the Amis of Formosa (36%) and the Filipinos of Mindanao (50%) as well as in the Kiritimati of Micronesia (70%), and Tonga and Samoa of West Polynesia (54% and 33%, respectively), and it drops to low frequencies in populations of East Polynesia. The communities of Polynesia and Micronesia exhibit considerable inter- and intra-population haplotype sharing suggesting extensive population affinity. The observed affinities, as well as the ages and diversity values within the P 164 sub-haplogroup among Austronesian-speaking populations signal an ancestral migration route and relationships that link the Amis of Taiwan with distant communities in West and East Polynesia, Micronesia, and the Maori of New Zealand. High resolution sequencing of the Austronesian Y chromosome indicate that the P 164 lineage originated about 19,000 ya and then split into three branches separating the Ami aborigines, Southeast Asian and Polynesian/Micronesian populations about 4700 ya, roughly coinciding with the initiation of the Austronesian diaspora. The Y-chromosomes of all the Polynesian and Micronesian population examined belong to the new FT 257096 haplogroup.

## Introduction

### Austronesian language speakers of Taiwan

Archeological, linguistic, and genetic data are routinely used to investigate ancestral relationships among human populations. Archaeological sites indicate affinities between southern China and Taiwan, suggesting the first settlers of Taiwan migrated from coastal Mainland Southeast Asia (MSEA)^1^. Specifically, Southeastern China has been identified as the source-region of Neolithic crops transported overseas to Taiwan and subsequently to Island Southeast Asia (ISEA)^2^. Archaeological evidence also provides evidence that several independent migrations from various coastal locations of MSEA populated different regions of Taiwan^3^. It has been suggested that the Dapenkeng culture (TPK culture) and the Lapita Cultural Complex (LCC) originated in farming communities of MSEA and were then introduced to Taiwan and the Philippines^4^. The anatomically modern human (AMH) fossil known as Zuozhen Man and artifacts unearthed from the Cailiaoxi River Valley in the southwestern plains of Formosa near Tainan City have been dated to 20–30 thousand years ago (kya) and pinpoint to an occupation that extends to as recent as 7–6 kya^5^. The occupation of Changbinian groups of Eastern Taiwan and Baxiandong in the central east coast, dated to 15 to 5 kya, exhibit similarities with the people of Fujian Province of coastal southern Mainland China^6^. Several cultural traits shared by several Taiwanese aboriginal communities are seen in the Yüeh people of Southern China and Northern Vietnam^7^. It has been suggested that pre-Austronesians expanded south along the coast from Shandong (coastal northeast mainland China) ~ 7 kya to reach northwest Taiwan ~ 4 kya^8^.

Linguistic studies add support to the theory that multiple migrations from MSEA populated Taiwan. The Austronesian languages spoken by Taiwanese aborigines are phonologically and lexically distinct and all are mutually unintelligible^9^. The Formosan vernaculars do not cluster into clades suggesting their old and unique origins^10^. The linguistic diversity of Taiwanese aboriginal languages provides ancestral links to the Austronesian-speaking populations of the Philippines and beyond into Oceania. In addition, the internal homogeneity, and the high degree of genetic diversity among the Taiwanese tribes also suggest their ancient and independent origins^11,12^. Currently, all the Austronesian-speaking aboriginal communities of Taiwan are culturally, linguistically, and genetically distinct^12^*.*

### Dispersal route and timeline of Austronesian language speakers

The Austronesian dispersal across the Pacific Ocean is thought to have occurred within a relative short time. This dynamic migration of Austronesian speakers out of Taiwan that extended into Oceania is referred to as the “Express Train” theory and the “Out of Taiwan” hypothesis, respectively, and is supported by archeological, linguistic, anatomy (dental and cranial metrics), and genetic evidence from the islands of Micronesia, Indonesia, Malaysia, Melanesia, and Polynesia^13^. The Austronesian dispersal took place along the shorelines of the islands of the Philippines, Indonesia, Melanesia, and Micronesia and culminated when the migrants reached and settled the remote islands of East Polynesia, Hawaii and New Zealand in the Pacific Southwest^1^. It is theorized that Austronesian speakers started migrating south from Taiwan ~ 5 kya^14^. It is thought that the Austronesian-speaking migrants experienced a layover of ~ 1–2 ky in Taiwan as they developed the necessary technology and skills necessary for crossing the dangerous open ocean between Taiwan and the Batanes Islands of the Philippines^15^. The Philippines is envisioned as the dispersal center of the Austronesian dispersal^16^. From the Philippines, Austronesian speakers proceeded in a southeasterly direction, sequentially colonizing archipelagos in Malaysia and Indonesia. This route delineates a costal path that resulted in the settlement of Melanesia and Micronesia ~ 3500 ya^17^. Genetic markers indicate that in Malaysia and western Indonesia, Austronesian speakers confronted the original settlers that populated the region during the original Out of Africa migration, allowing for various degrees of admixture^18^. Austronesian speakers migrated along the northern coast of New Guinea and nearby archipelagos in an easterly direction reaching the islands of the Bismarck, Solomon, Santa Cruz, and the Vanuatu Archipelagos^19^. Subsequently, the Tonga and Samoa Archipelagos were settled ~ 3.3 kya and ~ 3 kya, respectively^20–22^. According to radiocarbon dating, the Society Islands of East Polynesia were colonized ~ 1 kya^23,24^, while the Marquesas were settled ~ 830–730 ya and Rapa Nui (Eastern Island) ~ 820 ya, Hawaii 800–850 ya, and New Zealand ~ 740 ya ^23,25^.

### The O2a2b-P164 sub-lineage

Just over a decade ago sub-haplogroup O2a2b-P164 (formerly O3a2c-P164) was described as a link between the Ami aborigines of Taiwan and the populations of Tonga and Samoa suggesting that this native community from Formosa was a source population of the Austronesian speakers that settled West Polynesia^26^. Subsequently, it was established that O2a2b-P164 is widely distributed in the eastern coastal regions of Asia, from Korea to Vietnam and signals a genetic connection between proto-Austronesians-speaking populations of MSEA and the early Austronesian-speaking populations that crossed the Taiwan strait ~ 4800 ya and then spread into Oceania^27^. According to this study, these Austronesian migrants were the ancestors of the Neolithic farmers that cultivated rice and foxtail millet in northern Taiwan ~ 4500 ya ^28^. More recently, the links between this haplogroup was further delineated when the sub-lineage O2a2b2a2b-B451 was identified as the genetic connection between ancestors of Austronesians speakers of ISEA and Oceania and ancient groups from North China^27^. The O2a2b2a2b-B451 sub-lineage was also found to be present in Austronesian-speaking populations^27,29^ and to represent the best patrilineage genetic marker signaling a contribution from the Austronesian-speaking diaspora to ISEA^27^. Individuals positive for this sub-lineage in the Society Islands of Easter Polynesia were also typed positive for the downstream O2a2b2a2b1-B450 marker sharing a most recent common ancestor (MRCA) at ~ 5700 BP with a Sama-Bajaw individual from Sulawesi Islands in East Indonesia^27^. This body of evidence suggested to us that the P 164 mutation may represent an informative genetic marker for delineating the Austronesian dispersal.

### Aims of the study

Considering the many questions that remain unanswered pertaining to the routes and timing of the Austronesian dispersal, in this study, we explore in more detail the Y-chromosome sub-haplogroup O2a2b-P164 in Austronesian-speaking populations. This article describes, for the first time, the continuity of the Austronesian dispersal from the Amis of Taiwan throughout Micronesia and West, East and Southwest Polynesia as reflected in the distribution and diversity of P 164 individuals using high-resolution sequencing of Y chromosomes. These approaches uncovered a high-resolution lineage, FT 257096, within sub-haplogroup O2a2b-P164 that specifically signals the Polynesia dispersal from ISEA to Micronesia and the fringes of East and Southwest Polynesia.

## Materials and methods

### Declarations

All samples and Y-STR data enumerated in Supplementary Tables 1 and 2 were obtained from the published literature. Written informed consent was obtain from all samples sequenced by FamilyTreeDNA for the public domain information provided to the company and used in the Time Tree. These samples are the Fiji (n = 1), New Zealand (n = 3), Niue (n = 1), Papua/New Guinea (n = 2), Samoa (n = 2) and Tonga (n = 2). For these samples written informed consent was acquire voluntarily while closely adhering to the ethical guidelines stipulated by Colorado College, Colorado Springs, Colorado USA and FamilyTreeDNA. The study adhered to the tenets of the Declaration of Helsinki for the protection of human subjects. The IRB of Colorado College and FamilyTreeDNA approved this study. All experimental protocols were approved by the IRB of Colorado College and FamilyTreeDNA.

### DNA extractions and storage

DNA extraction from swabs of 6 samples 6 from Taiwan (n = 2, TW 102 and TW 106), Kiritimati (n = 3, TARA 89, TARA 97 and TARA 134) and the Marquesas (n = 1, NH 10 ) was performed using the standard phenol–chloroform method and ethanol-precipitated as described previously^13^. A NanoDrop 1000 Spectrophotometer (Thermo Scientific) was used for DNA quantitation. Samples were stored as stock solutions in 10 mM Tris–EDTA at − 80 °C.

### Haplotyping and sequencing of samples

**Figure 1:**
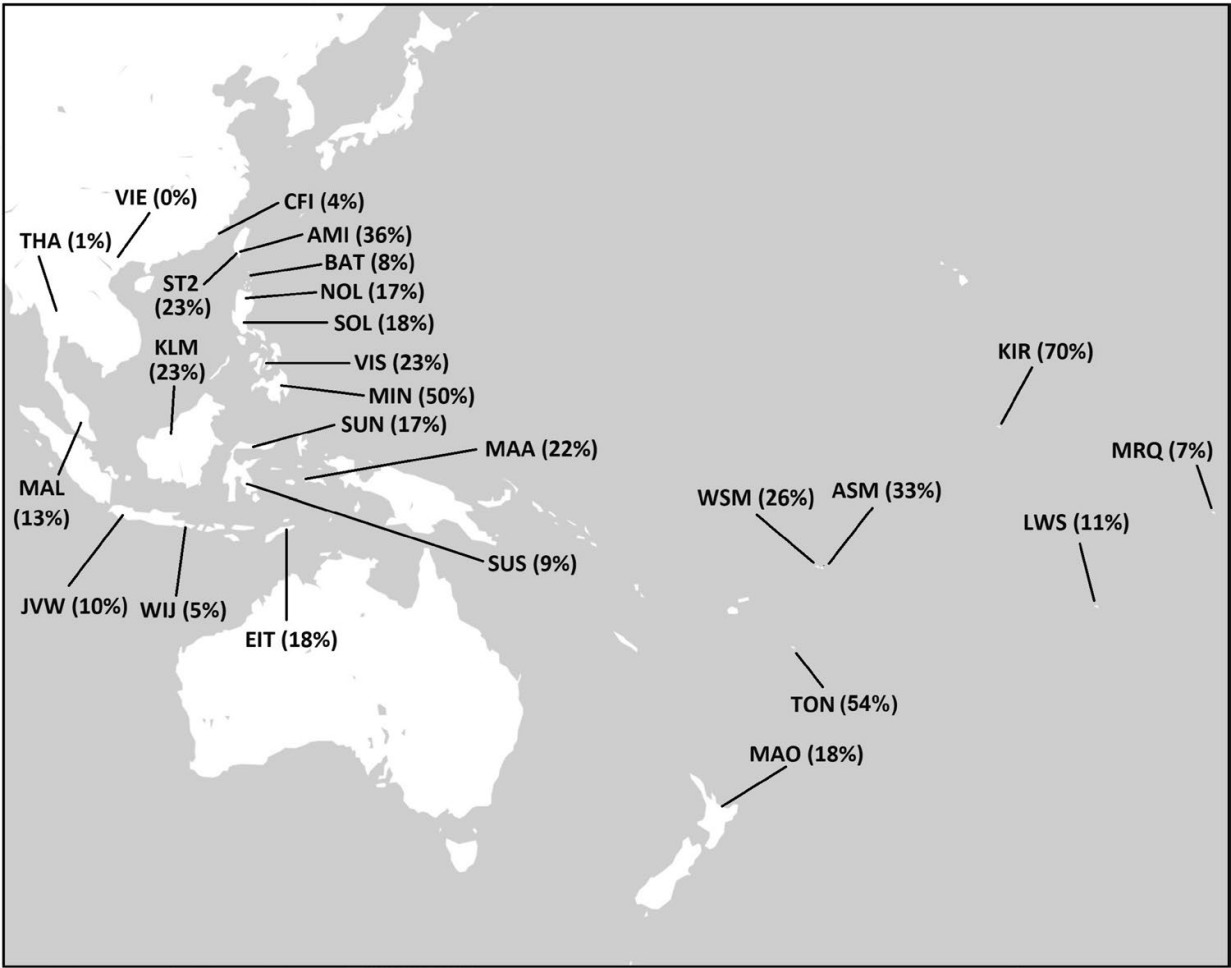
Geographical and frequency distribution of haplogroup O2a2b-P164. The list of populations and their codes are in Supplementary Table 1. Percentages in parenthesis are the haplogroup O2a2b-P164 frequencies in each polulation.

A total of 427 DNA samples from MSEA, ISEA, Micronesia and Polynesia with a O2a2b-P164 background were previously typed for 17 Y-STR loci (DYS19, DYS385a/b, DYS389I, DYS389II, DYS390, DYS391, DYS392, DYS393, DYS437, DYS438, DYS439, DYS448, DYS456, DYS458, DYS635, GATA H4) using the AmpFlSTR Yfiler kit (Applied Biosystems, Foster City, CA). The populations examined, number of individuals and references to the previously published populations used in the phylogenetic analyses are presented in Supplementary Table 1. The geographic locations of the examined populations are illustrated in Fig. 1. The phylogenetic relationship of the O2a2b-P164 sub-haplogroup to related sub-haplogroups are included in Supplementary Fig. 1. The most current version of the Y-DNA Haplogroup Tree was employed and can be found at http://www.isogg.org/tree/ (International Society of Genetic Genealogy. 2020. Y-DNA Haplogroup Tree 2020, version: 15.73. 11 July 2020).

From the samples which Y-STR haplotypes appear in Supplementary Tables 1 and 2, a total of 6 individuals from Taiwan (n = 2, TW 102 and TW 106), Kiritimati (n = 3, TARA 89, TARA 97 and TARA 134) and The Marquesas (n = 1, NH 10) were selected for high resolution sequencing by Big Y (FamilyTreeDNA). Supplementary Table 2 lists their codes. The procedures utilized are describe in Begg et al.^30^. Briefly, the samples were sequenced using the Illumina NovaSeq 6000 platform following Y chromosome capture with a proprietary capture protocol available at FamilyTreeDNA using the commercially available Big Y-700 service. The targeted enrichment design utilizes 155,000 capture probes for sequencing the non-recombining male-specific parts of the Y chromosome to high coverage (approximately 15–17 Mbp with 35–105 × depth, depending on sample quality). The human reference GRCh38 version was used for the next generation sequence analysis. In addition, public domain information derived from the genetic analysis of customers that used FamilyTreeDNA genotyping services were employed. These public domain samples are Fiji (n = 1), New Zealand (n = 3), Niue (n = 1), Papua/New Guinea (n = 2), Samoa (n = 2) and Tonga (n = 2).

### Phylogenetic and diversity analyses

Y-chromosome STR haplotypes under sub-haplogroup O2a2b-P164 (Supplementary Table 2) from a total of 46 previously published, geographically targeted, populations (Supplementary Table 1) were chosen for phylogenetic comparisons. The degree of haplotype reiteration for each population is indicated in Supplementary Table 3.

A Multidimensional Scaling (MDS) analysis was performed at the level of populations (SPSS v.20) using Rst distances estimated from haplogroup O2a2b-P164 Y-STR haplotypes^31^. The pairwise population comparisons were tested at a significance level of 0.05 with 10,000 permutations. To compensate for potential inclusion of false positives, type I statistical errors, the Bonferroni correction was applied (α/m = 0.05/136 = 0.0004). DYS385 was excluded from the haplotype diversity calculations because it is not possible to discriminate between the DYS385a and DYS385b loci with the Y STR kit. The number of repeats at DYS389II was calculated by subtracting the number of repeats at DYS389I.

Median-joining (MJ) networks^32^ based on Y-STR profiles of individuals possessing the P164 mutation were constructed with the NETWORK 4.5.1.6 software (www.fluxus-engineering.com), in which the Y-STR markers were weighted inversely to their repeat variance. Average gene diversity (GD) based on Y-STR loci were computed according to Nei^33^. Intra-haplogroup diversity (mean microsatellite variance: Vp)^34^ was calculated across 15 loci for each population. Only complete haplotypes (all 15 loci present) were used to compute Vp values. Y-STR haplotypes were used to estimate the time to the most recent common ancestor (TMRCA). With this aim, rho statistic (*ρ*)^35^ and weighted rho (*ρ*_W_)^36^ were estimated with an R script available in GitHub (http://github.com/fcalafell/weighted_rho). Mutation rates were obtained from the Y-Chromosome STR Haplotype Database (YHRD, www.yhrd.org) in March, 2023. Briefly, the weighted rho (ρw) method dates the haplogroups from STR variation with a weighted version of ρ that leverages on the relatively precise knowledge of the mutation rate of each Y-STR. It considers that mutations at slow STRs take longer to accumulate than mutations at faster STRs. For the calculation of rho (ρ), we used mutation rates available in YHRD as of 2023-07-03. On the Kilin-Klyosov TMRCA calculator, we assumed the age values given by the KKK (quadratic) approach. Calculations in the KKK (quadratic) approach are based on the discrete random walk numerical model, known in mathematics. The statistical significance of the time estimate differences was assessed using the Past 4.02 software (http://palaeo-electronica.org/2001_1/past/issue1_01.htm).

For the time tree, age estimates, branch points as a function of time, lineage and separation time of Y chromosomes estimated by high resolution re-sequencing were performed according to Begg and colleages^30^. The time tree based on Y chromosome high resolution re-sequencing of research and customers samples were done using a combination of automated shared variant detection and manual curation^30^. Briefly, the time tree diagram uses TMRCA estimates as calculated by Begg et al. 2023. It visualizes haplogroups (tree nodes) and samples on a timeline. It employs SVG, Javascript, and D3.js (https://d3js.org/) for its layout. On the vertical axis, each node and sample occupy a separate row, starting from the top left. The horizontal axis represents years ago (ya), with nodes positioned based on the estimated TMRCA (Time to Most Recent Common Ancestor) of their haplogroup. Nodes have 95% confidence bars and are shown in blue. Present-day samples linked to each haplogroup are arranged below it, ordered by their birth years (rounded for privacy), and depicted with country flags and name based on their self-reported patrilineal ancestry or an "Unknown" icon for unreported ancestry. Ancient DNA samples are placed next, represented by brown icons, and ordered by their dating, either radiocarbon or from archaeological contexts, with the uncertainty in the dates represented by confidence bars. Child nodes of each haplogroup are then displayed, with the youngest TMRCA estimates first, connected to their parent nodes. The diagram prioritizes minimal line crossing by rendering younger samples and nodes before older ones.

## Results

### Frequency and diversity distribution of sub-haplogroup O2a2b-P164 in MSEA, ISEA, Indonesia, and West, East and Southwest Polynesia

**Figure 2:**
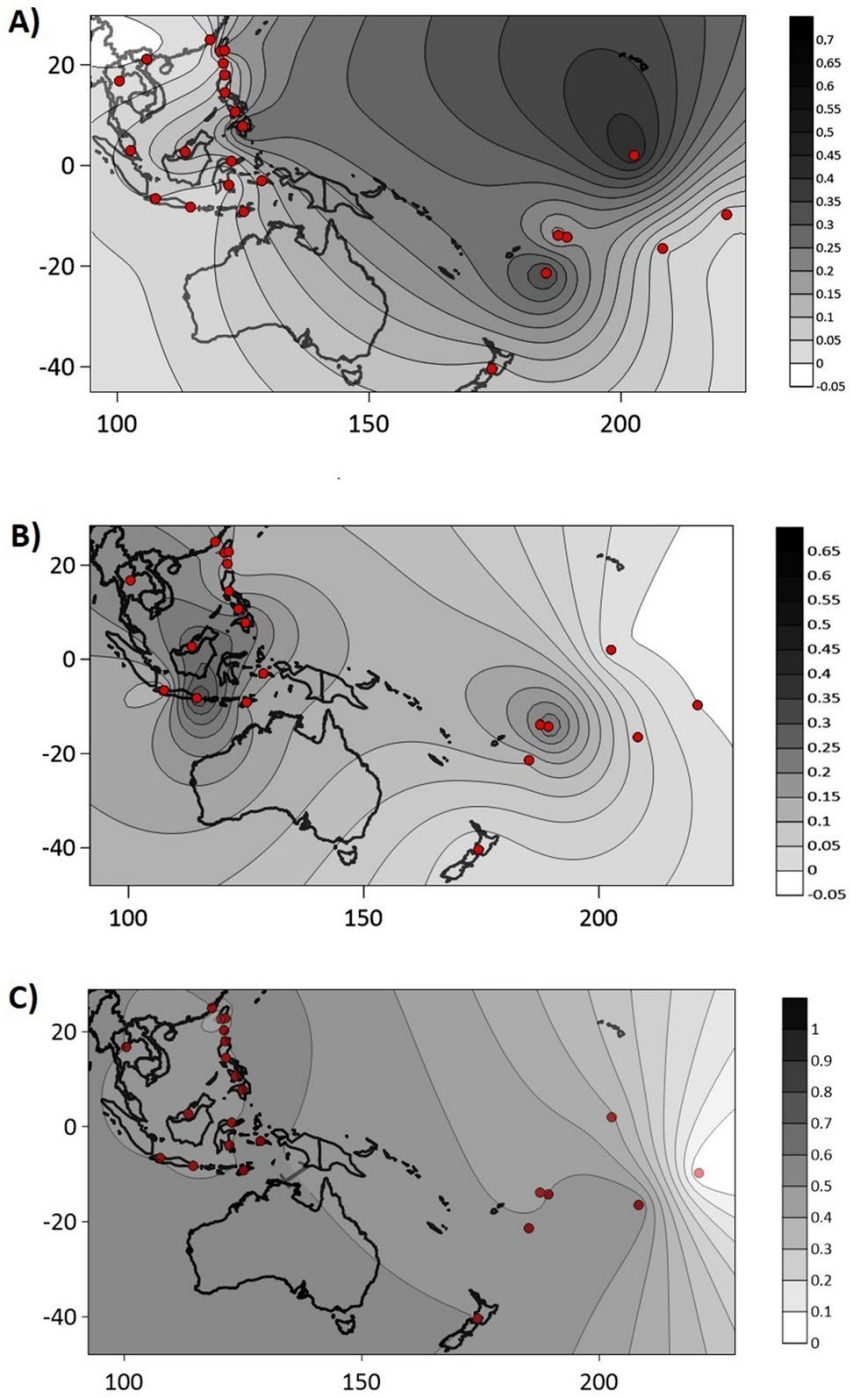
Distribution of O2a2b-P164 haplogroup frequencies and diersity values. The X and Y axes represent longitude and latitude respectively. The scales on the rightr side of the plots represent haplogroup frequency (**A**), Vp diversity (**B**) and Nei's diversity (**C**) values. The list of populations and their codes are in Supplementary Table 1.

Considering the wide presence of sub-haplogroup O2a2-P164 among MSEA, ISEA and Indonesian populations^37^, we undertook an exploration of its distribution in Micronesia^13^, West^26^, East^38^ and Southwest Polynesia^39^ (see Fig. 1 for location and frequency distribution). Contour maps were constructed to illustrates the distribution of frequencies and diversities in Near and Far Oceania (Fig. 2, panels A, B and C). The partitioning of populations within the frequency contour map traces a geographical arc from Taiwan south into the Philippine Archipelago, Borneo, Indonesia, and West and East Polynesia. The frequency of sub-haplogroup O2a2-P164 is abundant (36%) in the Ami aborigines of the east coast of Taiwan and exhibits a general decrease in the Philippines (with the notable exception of Mindanao Island) and West and East Indonesia (Fig. 1). This general frequency diminution continues eastward towards Oceania. This overall drop in abundance is interrupted in the islands of Kiritimati (Micronesia), Tonga and Samoa of West Polynesia, which displays abundant levels of 70%, 57% and 33% sub-haplogroup O2a2-P164, respectively.

The 17-loci haplotypes of the P 164 individuals examined in this study are provided in Supplementary Table 2. The number of reiterated haplotypes is non-existent in MSEA, Philippines and Indonesia, but present in West Polynesia and dramatically increase in East Polynesia. Two methods were utilized to access the genetic diversity of Y-STR haplotypes within sub-haplogroup O2a2-P164 in the populations. We estimated Nei’s and Vp diversity values (Supplementary Table 3) and contour maps were generated to illustrate the diversity distributions (Fig. 2, panels B and C). Both methods exhibit the higher diversity values in populations from MSEA as well as West and East Indonesia followed by groups from the Philippines Archipelago and then by the Siraya and Ami aborigines of Formosa. Except for the Vp diversities in American Samoa as well as the Society Islands, Tonga and the Maori using Nei’s diversity, the populations of Kiritimati in Micronesia as well as West and East Polynesia register the lowest diversity values with both estimation methods.

### Population relationships based on sub-haplogroup O2a2-P164

**Figure 3:**
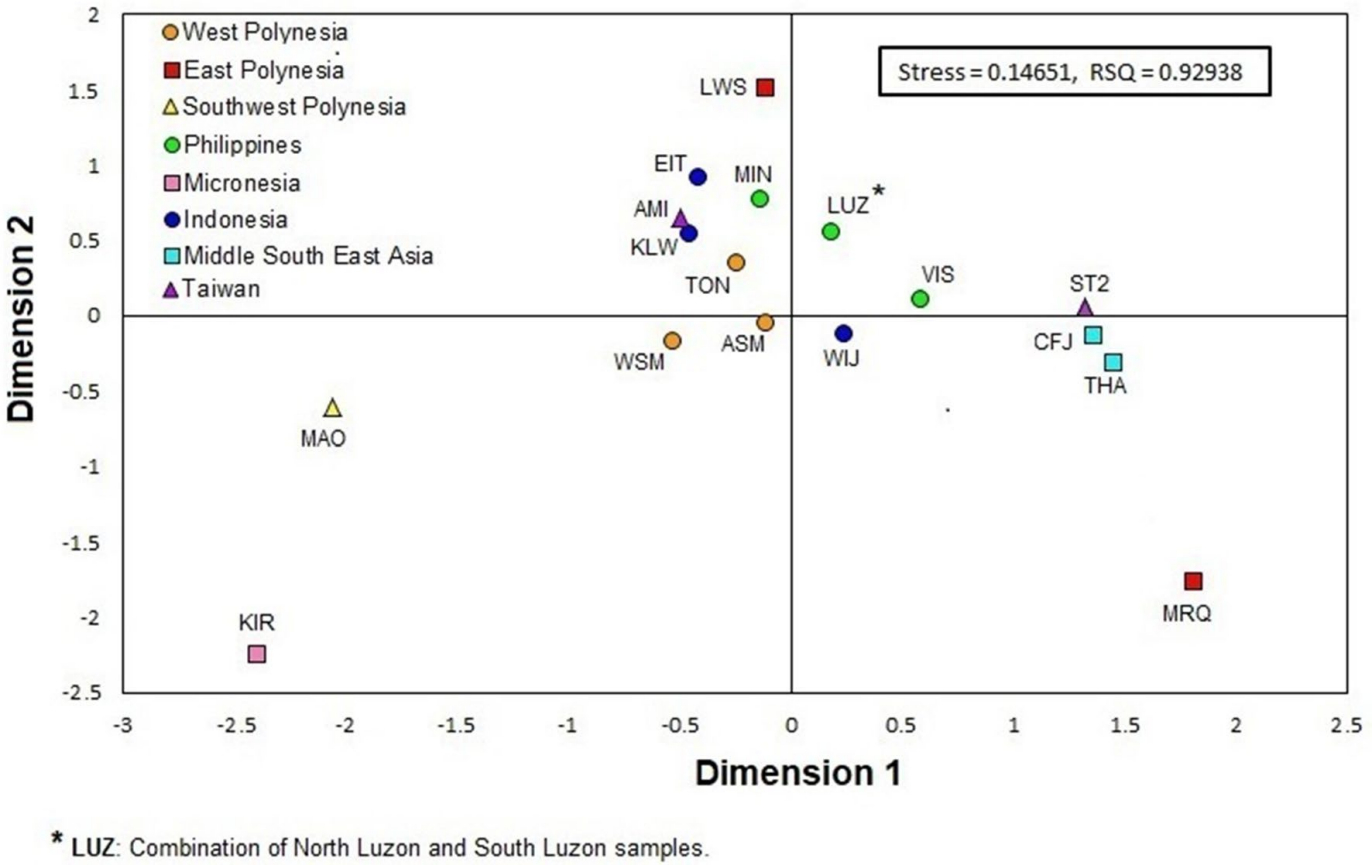
MDS plot of O2a2b-P164 haplogroup populations based on Y-STR Rst genetic distance. The list of populations and their codes are in Supplementary Table 1.

Rst pairwise genetic distances among the populations examined were calculated based on 15-loci Y-STRs haplotypes and are provided in Supplementary Table 4. The Rst distances with their corresponding p-values indicate that the populations of the Samoa Archipelago, especially American Samoa, share genetic affinities based on their Y-STR profiles under P 164 with all populations examined, especially from West Polynesia. Conversely, Kiritimati of Micronesia displays statistically significant differences (< 0.0001) with all of the other populations examined except the Maori. The distances and partitioning of populations based on the Rst values are illustrated in a MDS plot (Fig. 3). In this two-dimensional graph (stress value = 0.14651, R^2^ value = 0.92938), most of the groups from Oceania cluster at the center where the coordinates meet. Within this central aggregate some geographical partitioning is observed, mainly the nearness among the populations from West Polynesia as well as the relative proximity among the groups from the Philippine Archipelago. The two populations from MSEA (i.e., Han and Thailand) partition close to each other, some distance from the central cluster in quadrant IV. Noteworthy is the distant segregation from each other, and the central aggregate, of the Marquesas and Society of East Polynesia, the Maori of New Zealand, and Kiritimati of Micronesia as outliers. These groups are located at the geographical fringes of the Polynesian domain.

### Network analysis

**Figure 4:**
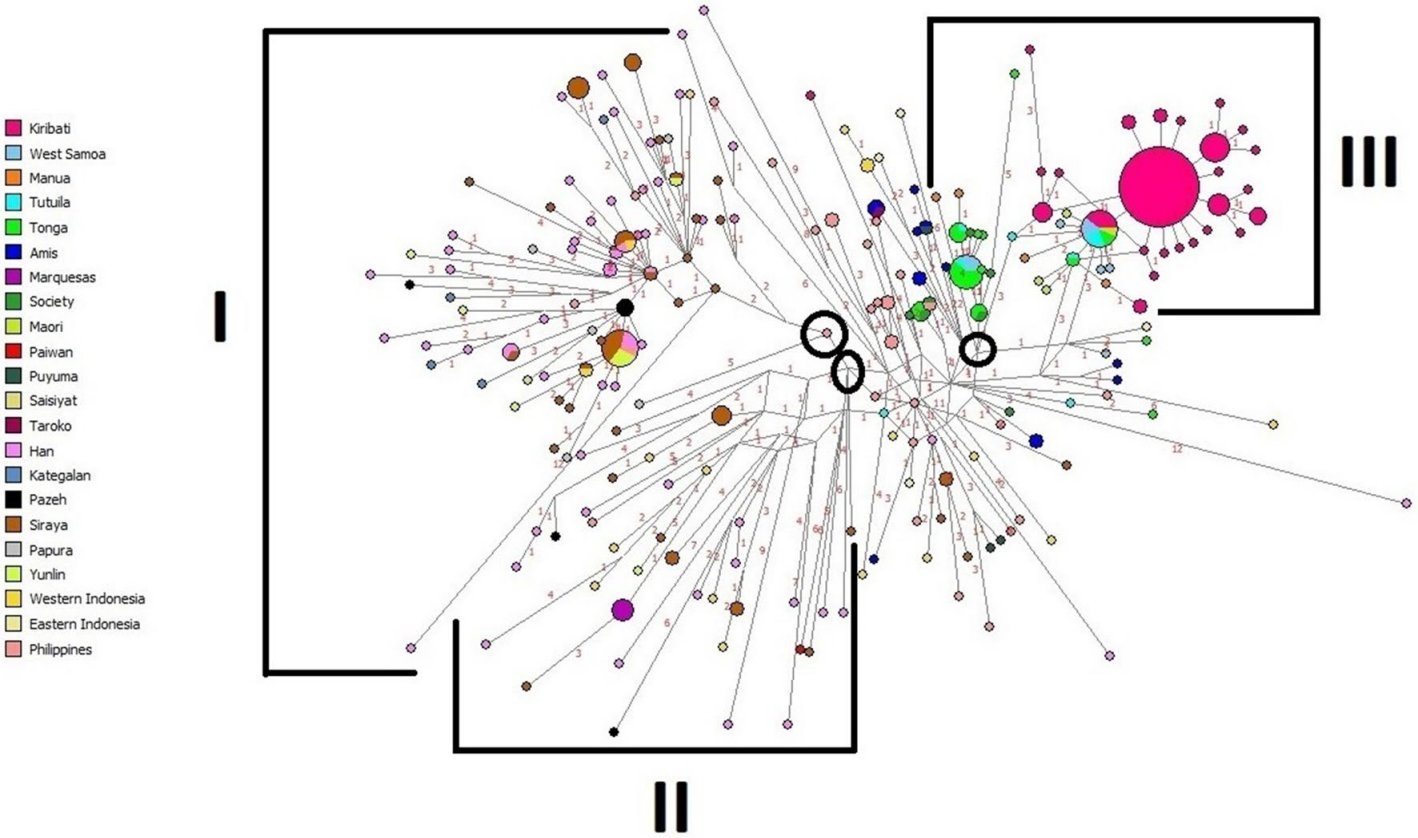
O2a2b-P164 haplogroup Y-STR haplotype network based on 15 Y-STR s. The list of populations, their codes and their groupings are in Supplementary Tables 1 and 2. Circles represent a haplotype. The size of the circle is proportional to the sample size. The length of the lines is proportional to the number of differences between haplotydes. Nodes without circles represent medial vectors which are hypothetical haplotydes/nodes which connect one haplotyde to the next. Black circles mark the roots of subgroups I, II and III.

The network analyses performed are based on 15-loci Y STR haplotypes within sub-haplogroup O2a2-P164. Figure 4 illustrates the relationships of haplotypes of populations from MSEA, Taiwan, Philippines, Western Indonesia, Eastern Indonesia, Near Oceania and Remote Oceania. These regions include populations of Hans, Melanesians, Micronesians, and Polynesians. Three groupings are discerned within the network. Groups I and II are represented by individuals from throughout ISEA, MSEA and Western Indonesian, mainly as singletons of Han, Sirayan (southwestern plains of Taiwan), Yunlin, Filipino and Western Indonesian populations. A prominent node made up of individuals from Kiritimati makes up the bulk of Group III with West Samoans, Tongans and Maori emanating from it as a smaller multipopulational satellite node. Group III also exhibits individuals from Tonga, Ami tribe (aboriginal Taiwanese tribe from southeast coast) and Tutuila (West Samoa). Overall, intra- and inter-population sharing of haplotypes is limited within group I and II. Group I exhibits only four inter-population nodes. Groups I and II do not show a spider-like topology and are mainly made of randomly distributed, singletons separated by 1–4 mutations and overall lack population sub-structure. Only group III illustrates a spider-like morphology and population substructure. In Fig. 4, circles within the network delineate the main nodes that define group I, II and III. In between groups I/II and III a heterogeneous zone of populations made up of samples from various regions, mainly from the Philippines, MSEA and Western Indonesia, exhibits multiple nodes and reticulations.

**Figure 5:**
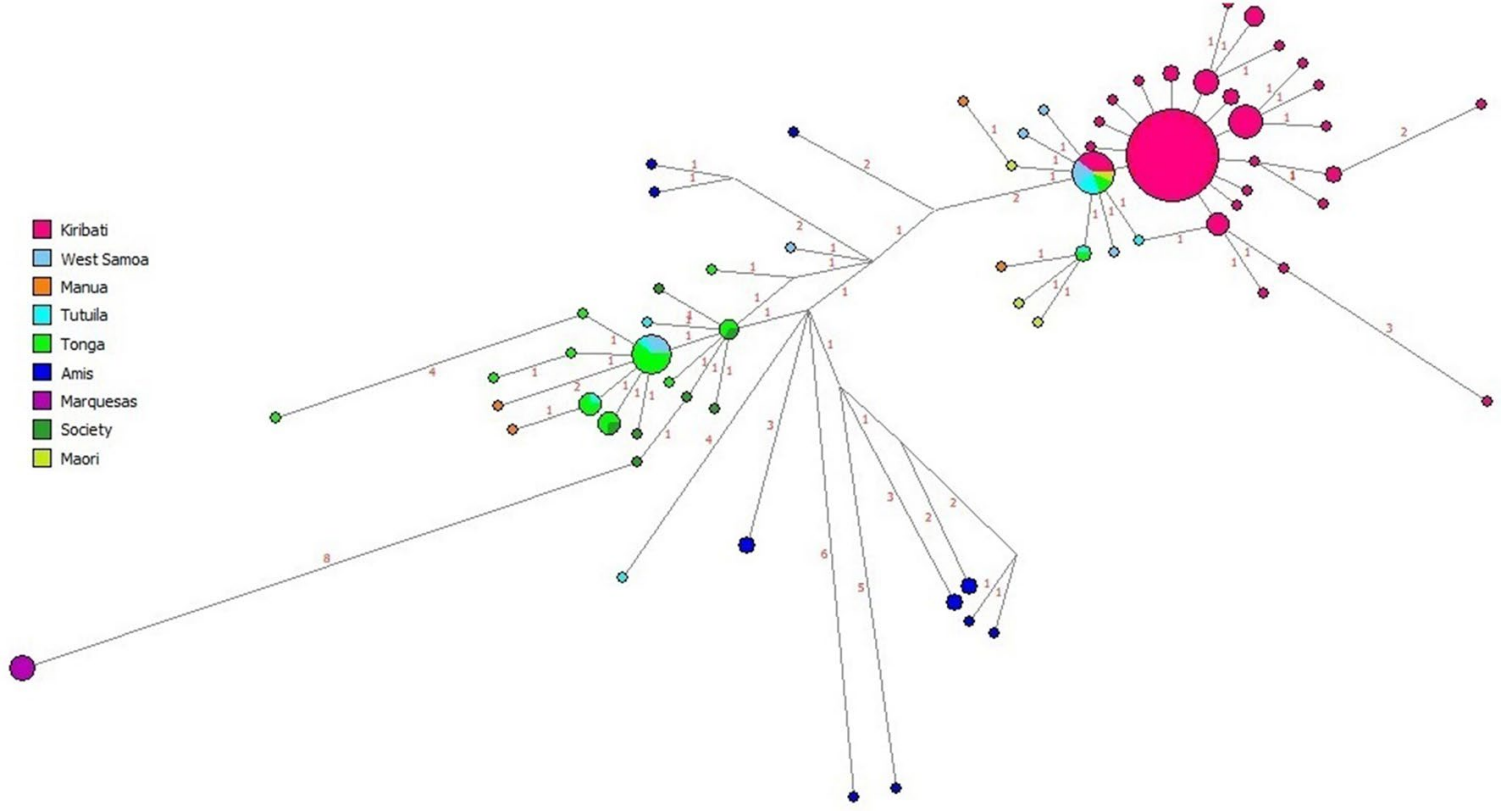
O2a2b-P164 haplogroup Y-STR haplotype network for Polynesia and Micronesia based on 15 Y-STRs. The list of populations, their codes and their groupings are in Supplementary Table 1.

To examine in more detail the relationships among individuals within Polynesia and the Ami aborigines of Taiwan, a regional network analysis was performed (Fig. 5). This network partitions individuals from Micronesia, West, East, Southwest Polynesia, and the Amis of Formosa revealing a bipolar topology. Except for the Marquesan and two Ami haplotypes, most haplotypes in this network are separated by only one mutational step, suggesting close affinity among the individuals from the entire Polynesian domain and the Amis. Also, throughout this network, considerable interpopulation haplotype sharing is observed. In one branch of the network, samples from Kiritimati partition with males from Tutuila, West Samoa, Tonga and New Zealand (Maori). As with the East Asian/Indonesian/Polynesian network (Fig. 4), most of the Kiritimati samples are identical, forming a central prominent node in a star-like structure. Branching from this dominant node, a secondary smaller inter-population node that differs by only one mutation step is made up of Maoris, Tutuilans, West Samoans and Tongans. At the other branch of the network, Tonga, Tutuila (American Samoa), Manua (American Samoa), West Samoa, the Amis and all the Marquesan samples partition. In this second pole, the Marquesas samples are separated from the rest of the haplotypes by eight mutational steps and are directly linked to a lineage made up of Society individuals that share a haplotype with samples from Tonga. It is notable that the Amis partition in proximity to several individuals from Tonga and the Society Archipelagos. Some Ami haplotypes are only 4–6 mutational steps from Samoan, Tongan and Society haplotypes. Also noteworthy is the close affinity of the Maoris to West Polynesians and Micronesians, some sharing the same haplotype with the Maoris. Austronesian speakers are thought to have migrated first from West Polynesia to the Marquesas and from there to New Zealand, a total distance of about 9294 km.

### Time estimastes

Table 1 exhibits the coalescence time estimations based on the available 15-loci Y-STR haplotypes under sub-haplogroup O2a2-P 164 of populations with sufficient number of individuals. The time estimations were calculated from Y-STR genotypes generated in the original studies^13,26,37–39^. Due to the limitations and assumptions associated with the current calibrations of Y-STR mutation rates^40–43^, as well as the differences in methodology among studies, the dates generated in this study should only be taken as relative estimates for comparisons among the populations examined in this report.

**Table 1:** Estimates of the time to the most recent common ancestor (TMRCA) of haplogroup O-P164 populations.

Population and sample size	Years/generation	Rho				ASD	
		Age		Weighted age		Years	SD
		Years	SD	Years	SD		
West Samoa	25	1994	653	1227	489	407	153
[O-P164]	30	2393	783	1473	586		
n = 11		rho: 2.8182		rho: 1.7343			
Kiritimati	25	434	93	412	85	415	71
[O-P164]	30	521	111	495	102		
n = 114		rho: 0.614		rho: 0.5827			
Tonga	25	1104	228	815	186	768	244
[O-P164]	30	1325	274	978	223		
n = 25		rho: 1.56		rho: 1.1519			
Society	25	943	334	592	264	943	404
[O-P164]	30	1132	400	711	317		
n = 6		rho: 1.3333		rho: 0.8373			
Tutuila (American Samoa)	25	2201	458	1702	385	1940	711
[O-P164]	30	2642	550	2042	462		
n = 9		rho: 3.1111		rho: 2.4053			
Han, Fujian (China)	25	2547	425	3696	511	3517	627
[O-P164]	30	3057	509	4435	614		
n = 10		rho: 3.6		rho: 5.2233			
Amis (Taiwan)	25	2613	455	2552	441	3832	1005
[O-P164]	30	3135	546	3062	529		
n = 13		rho: 3.6923		rho: 3.606			
South Luzon (Philippines)	25	2972	510	3714	595	5390	864
[O-P164]	30	3566	612	4456	715		
n = 10		rho: 4.2		rho: 5.2479			
Thailand	25	3774	545	5933	683	5966	1623
[O-P164]	30	4529	654	7120	820		
n = 9		rho: 5.3333		rho: 8.3849			
Visayan Island (Philippines)	25	3963	530	6517	679	5966	1623
[O-P164]	30	4755	635	7821	815		
n = 10		rho: 5.6		rho: 9.2105			
West Indonesia [Java + Kalimantan + Sumatra]	25	4993	473	7080	557	10,314	2784
[O-P164]	30	5991	568	8496	669		
n = 18		rho: 7.0556		rho: 10.0055			

The list of populations, their codes and their groupings are in Supplementary Table 1 and 2.

Rho: R script "weighted age" F. Calafell.

*SD* standard deviation.

The oldest age estimates were observed in West Indonesia (Java, Kalimantan and Sumatra) (5991 ya, 8496 ya and 10,314 ya, rho statistic, weighted rho and the Kilin-Klyosov method, respectively). The second oldest dates were detected in Visayan Island, Philippines (4755 ya, 7821 ya and 5966 ya, respectively), South Luzon, Philippines (3566 ya, 4456 ya and 5390 ya, respectively) and Thailand (4529 ya, 7120 ya and 5966 ya, respectively). Next in longevity is seen in the Hans of Fujian province, across the Strait of Taiwan, mainland China (3057 ya, 4435 ya and 3517 ya, respectively). The Amis of Taiwan follow closely with age estimates of 3135 ya, 3062 ya and 3832 ya, respectively. The Samoan and Tongan archipelagos of West Polynesia register much recent dates (1,473–2,042 ya and 978 ya, weighted rho, 30 years/generation, respectively) while in the Society Islands of East Polynesia, the sub-haplogroup O2a2-P 164 dates from 711 ya (weighted rho, 30 years/generation) to 943 ya (Kilin-Klyosov method). This distribution of dates illustrates a cline of decreasing ages that start in Southeast Asia, crossing into Taiwan and then dispersing into West and East Polynesia. The very recent age of 495 ya (weighted rho, 30 years/generation) and 415 ya (Kilin-Klyosov method) of the P 164 mutation in the island of Kiritimati suggest a post-Austronesian dispersal introduction of the P 164 mutation into this region of Micronesia.

### Sequencing of the Y chromosome

**Figure 6:**
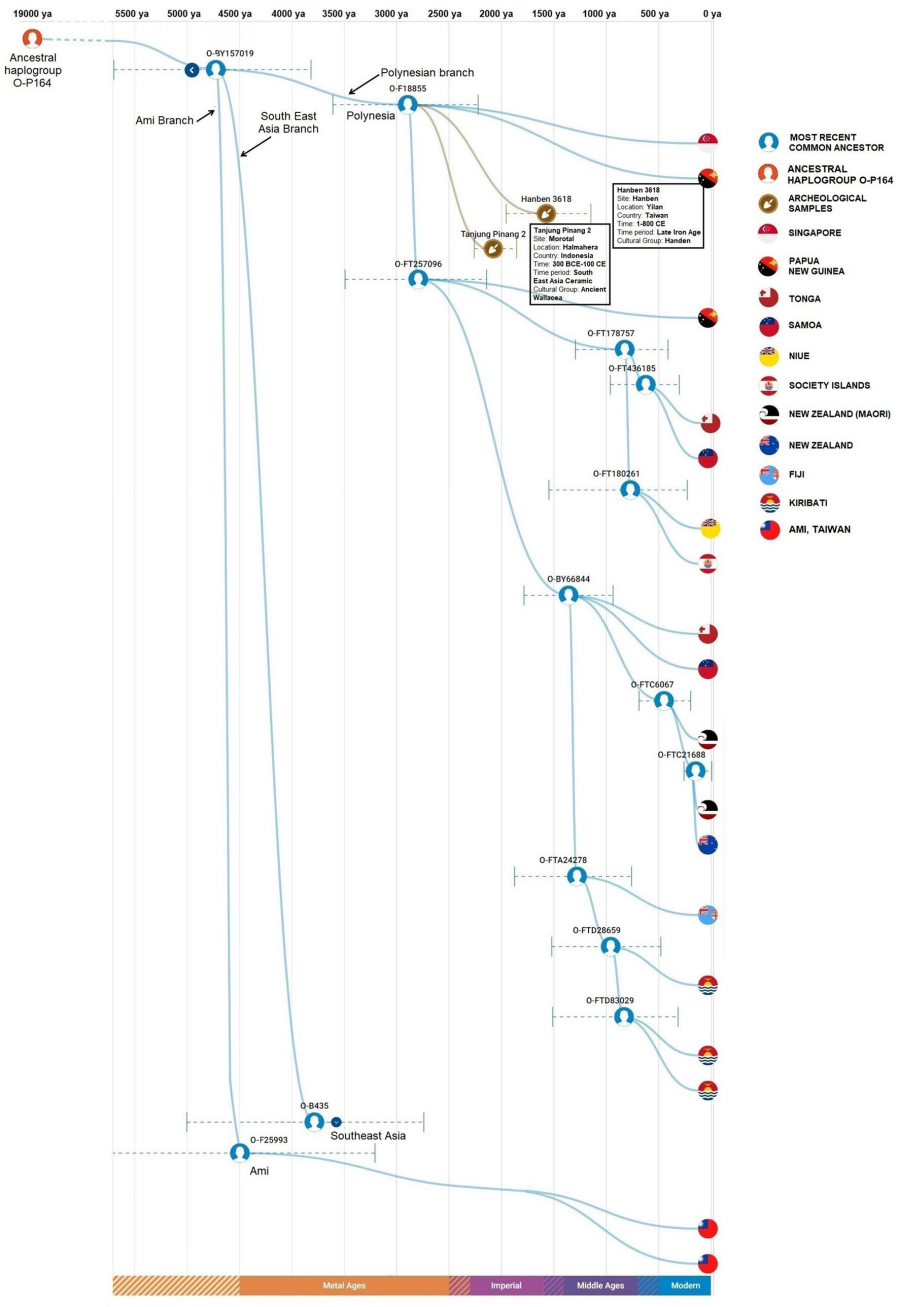
Sub-haplogroup P164 tree based on Y-chromosome sequences. O-B435, O-F25993, O-F18855 and other codes like these refer to haplogroups.

To delineate further the ancestry of the O2a2-P164 sub-haplogroup in Austronesian speakers, we undertook the high-resolution sequencing of Y chromosomes of selected samples of individuals from Taiwan (Amis) and Oceania. This information was supplemented with publicly available Y-chromosome data from customers of Big Y service (FamilyTreeDNA). Figure 6 represents a Time Tree of Y-DNA sub-haplogroup P164 illustrating the segregation of three branches representing Southeast Asian, Amis and Polynesian individuals. These three branches share a common ancestor and founder of the P 164 mutation who lived ~ 19,000 years ago (ya). The Southeast Asian, Amis and Polynesian branches represent three lineages that have been assigned specific sub-haplogroup designations, O-B435, O-F25993 and O-F18855, respectively. The split of the Southeast Asian, Amis and Polynesian branches occurred ~ 4700 ya from a hypothetical common ancestor designated O-BY157019. The two lineages corresponding to the two re-sequenced Ami individuals separated ~ 2000 years ago (ya), likely in Taiwan. The separations of lineages of individuals from Singapore and Papua from Polynesians are thought to have occur ~ 2850 ya. These splits occurred from common ancestors O-F18855 and O-FT257096. Although haplogroup O-F18855 exhibits males from outside the Polynesian domain (*e.g*., Singapore and Papua New Guinea), all re-sequenced samples from Polynesia and Micronesia, except one from the Marquesas, partition within this sub-haplogroup (Fig. 6). Some sub-structure is evident in this Time Tree with the Maori individuals clustering by themselves within the O-FTC6067 branch and the Fijians with the Kiritimati samples within haplogroup O-FTA24278. These branches had a common ancestor that lived ~ 1300 ya. The Fijian and the Kiritimati branches separated ~ 1200 ya likely in Fiji and the splits among the Kiribatian branches occurred ~ 900 and ~ 800 ya, likely in the Gilbertese Archipelago of Micronesia. The Tongans and Samoans of West Polynesia segregate together and the sample from the Society Islands partition with Niue (West Polynesia). Two archeological samples from Yilan, Taiwan and Halmahera, Indonesia corresponding to the Hanben (1–800 CE) and Ancient Wallacea (300 BCE–100 CE) cultural groups, respectively, branch off from the roots of the Polynesian branch O-F18855. It is likely that these two archeological samples may represent part of the Austronesian dispersal that eventually populated the rest of the Pacific.

It is notable that the single re-sequenced sample from the Marquesas of East Polynesia did not group with the other Polynesian samples as part of the FT257096 sub-haplogroup. The Marquesas sample branched off from the P 164 lineage close to the sub-haplogroup’s origin ~ 19,000 ya. The Marquesas lineage forms a branch with two Central Thai samples^44^ that in turn diverged from the Marquesas lineage ~ 5500 ya. It is likely that The Marquesan and Thailanders branch had a common ancestor in MSEA.

## Discussion

### Continuity of sub-haplogroup O2a2b-P164 from MSEA into Far Oceania

The frequency distribution of P 164 from coastal MSEA into East and Southwest Polynesia demonstrates that a component of the male Austronesian-speaking migrants were carriers of this mutation. P 164 persisted in these voyagers as they discovered and settled all the Pacific islands that we examined, including the Gilbertese Archipelago in Micronesia. The distribution of this sub-haplogroup indicates that its presence is low in MSEA where it probably originated^27^ ~ 19,000 ya and subsequently experienced an increased in some of the indigenous groups that migrated into the island of Taiwan ~ 7500 to 7900 ya^45^. Currently, the Amis (36%) and the Siraya (23%) of southeast and southwest Taiwan, respectively, exhibit appreciable frequencies of the sub-haplogroup O2a2b-P164 (Fig. 1). The other moderate level of P 164 (13%) is seen in Puyuma of the southeast lowlands of Formosa. The abundance of P 164 is in the low single digests or zero in the other major tribal groups of the island. In the Philippine Archipelago, the P 164 mutation frequency increases latitudinally from 8% in the northern Batan Islands of the Luzon Strait to 50% in the southern island of Mindanao. Drift was likely the force responsible for this clinal increase. In the islands of Western Indonesia, the frequencies remain in single digests or low 10s, experiencing a moderate increase in Eastern Indonesia (17–22%). In the islands of Samoa and Tonga of Western Polynesia and in Micronesia, P 164 increase dramatically to 26%/33%, 54% and 70%respectively. In East and Southwest Polynesia, the frequencies experience a drop, to single digit and low 10s in the former. These fluctuations are likely the result of random drift as migrants settled isolated islands and insular communities experienced limited subsequent communication with other islands. Additional comprehensive and more granular data from other insular populations should provide a better framework to access migration and communication among Oceanic islands. Also, some of the collections examined in this study contain limited number of samples. These collections are precious, and some are represented by small number of individuals. Nevertheless, greater number of individuals per population should improve the degree of certainty in the numbers generated.

An assessment of the diversity distribution based on Y-STR haplotypes among the same populations examined for P 164 frequencies provides a view of the genetic heterogeneity landscape within the sub-haplogroup as a function of geographical distance from its origin in MSEA. It also allows for comparisons of the abundance of the P 164 mutation and its relation to the diversity of the haplotypes within the sub-haplogroup. The Nei’s and Vp diversity values observed exhibit a general demic decrease in diversity as a function of geographical distance following the putative route taken by Austronesian speaking migrants during their dispersal. In instances of progressive sequence of migration events and colonization of relative isolated lands, diminution of diversity is expected. In the Near and Far Oceania diversity landscape seen in this study, we noticed only three instances in which Y-STR haplotype diversity was relatively high in regions where the rest of the populations exhibited the anticipated low values: the high Vp values in the Samoan Islands and the Nei’s diversity in the Society Islands, Tonga and Maoris. These relative high diversity values may have resulted from multiple migrations into the regions from different source populations and/or time periods. The regular well-documented trade routes among Polynesians islands may have facilitated gene flow and high levels of diversity^46^.

### Affinity among the Ami aborigines of Taiwan, Micronesians, and Polynesians

The Y-chromosome haplotype affinity landscape illustrated in the network analyses is congruent with the extensive and fast dispersal of the sub-haplogroup O2a2b-P164 throughout Near and Far Oceania (Fig. 4). The populations included within the large geographical expanse in the network include Hans, Filipinos, Malays, Indonesians, Melanesians, Micronesians, and Polynesians. This likeness is particularly reflected in the Y-STR haplotype uniformity under the P 164 mutation among Polynesian and Micronesian populations. It is notable that most of the non-Polynesian haplotypes are separated by multiple mutations, and partition rather randomly over most of the network mostly as singletons in branches lacking population structure. This type of haplotype distribution is indicative of limited genetic affinity. On the other hand, the haplotypes of West Polynesia, East Polynesia, Southwest Polynesia, Micronesia, and the Amis of Taiwan are represented by prominent nodes exhibiting inter- and intra-population haplotype sharing, separated (except for the Marquesas samples) by single mutational steps, suggesting extensive haplotype affinity throughout Polynesia and Micronesia (Fig. 5). This genetic homogeneity signals ancestral relationships that link one specific Taiwanese aboriginal population, the Amis, to distant populations such as the Austronesian-speaking groups in West and East Polynesia, Micronesians, and the Maoris of New Zealand (Fig. 5). It is likely that these close affinities stem from the rapid speed of the Austronesian dispersal as well as extensive trade throughout the Polynesian/Micronesian domain subsequent to colonization. It is also noteworthy that, as part of this long-distance Pacific affinities, a specific lineage links Amis, Tongan, Society and Marquesas (in that order) individuals in the regional network (Fig. 5). The linkage among these populations reinforces the notion of the specific origin and subsequent direction of the Austronesian dispersal.

### Sequencing of the Y chromosome

To discern the relationship of Amis, Polynesian and Micronesian P 164 Y chromosomes in more detail, we undertook the high-resolution re-sequencing of selected samples from Near and Far Oceania. Our exploration indicates that the P 164 lineage originated about 19,000 ya and then split ~ 4700 ya into three branches representing Amis, Southeast Asians, and Polynesia/Micronesian lineages (Fig. 6). The separation of these three branches coincides chronologically with the time Austronesian-speaking agriculturists departed from MSEA for Taiwan ~ 6000 ya to when they left from Formosa ~ 4000 ya to colonize Oceania^45^, yet closer to the initiation time of the dispersal. A split of these three population groups at this time is congruent with a putative migration wave that left Taiwan, moved south into ISEA and northeasterly into West Polynesia. A subsequent separation of the samples from Singapore and Papua from Polynesians as indicated by the Time Tree (Fig. 6) dates to ~ 2850 ya likely occurred in ISEA during the early stages of the Austronesian dispersal. Some sub-structure indicating the separation of Maori, Fiji and Kiritimati samples from other Polynesians ~ 1300 ya fits the putative timeline and direction of more recent colonization events. Our exploration of the sub-haplogroup O2a2b-P164 in the Pacific demonstrates that *all* the Polynesians and Micronesian individuals examined in the study, except the Marquesas sample, fall into a well-define sub- haplogroup, O-FT257096, which separated from the rest of P 164 haplotypes ~ 2700 ya.

Within the observed Y-chromosome homogeneity of P 164 individuals throughout Polynesia and Micronesia, we see the unique case of the Marquesas sample that partitions distantly in the network, whole Y chromosome high resolution resequencing and MDS analyses. In fact, the Marquesas Y chromosome branch dates to the time the P 164 mutation occurred ~ 19,000 ya. The Marquesas sample shares a branch with two Central Thai^44^ samples. It is not clear how the ancient Marquesas P 164 Y chromosome lineage, which likely originated in MSEA, ended up in extreme East Polynesia. Considering that Chinese migrants settled French Polynesia and specifically the Marquesas as laborers as part of the cotton industry in the mid nineteenth century^47^, it is possible that the P 164 Marquesan individual is a descended of recent indentured worker from mainland China and its descendants in fact were not part of the Austronesian dispersal.

## Conclusion

The partitioning of populations in the MDS plot, the affinities based on the Rst values, the topology of the networks, the frequency and diversity distributions and the high-resolution re-sequencing of the Y chromosome all are concordant and in agreement with the origin of the P 164 mutation in MSEA, its movement to Taiwan, and its dispersal into Oceania. Also, all these lines of evidence indicate that in MSEA, the Philippines and Indonesia a marked level of diversity within P 164 exists, while in the populations within Polynesia and Micronesia relative homogeneity within the sub-haplogroup predominates. It is likely that this geographical dichotomy in diversity within the O2a2b-P 164 sub-haplogroup stems from the compounded diminution in diversity resulting from serial founder effect events leading to genetic drift as migrants settled distant isolated islands sequentially. In some instances, this process has led to the predominance of specific Y-chromosome haplotypes under P 164 (e.g., 25% in Kiritimati). According to our exploration of the sub-haplogroup P 164 using Y-STR markers and high-resolution re-sequencing, the Austronesian dispersal move as a wave that started with the Amis of Taiwan moving fast, first south towards the Philippines and Indonesia and then north and east penetrating Polynesia and Micronesia. This dynamic process gave rise to several island communities exhibiting rather homogeneous P 164 Y chromosome populations as accessed by the limited diversity of their Y-STR haplotypes.

### Supplementary Information

Supplementary Figure 1.

Supplementary Table 1.

Supplementary Table 2.

Supplementary Table 3.

Supplementary Table 4.

## Data Availability

All the data utilized in this study, which includes the 17-loci Y-STR haplotypes within the O2a2b-P164 background of all populations examined are provided in Supplementary Table 2. The high-resolution re-sequencing data generated from the 6 individuals previously indicated were deposited at the European Nucleotide Archive under the study accession number PRJEB65754 and sample accession numbers ERS16310861- ERS16310866 and are publicly available as of the date of publication.
