# Supplementary figures and images for: Delineating the dispersal of Y-chromosome sub-haplogroup O2a2b-P164 among Austronesian-speaking populations
Source: Sci Rep. 2024 Jan 24;14:2066. doi: 10.1038/s41598-024-52293-z (PMC10808098; doi:10.1038/s41598-024-52293-z)

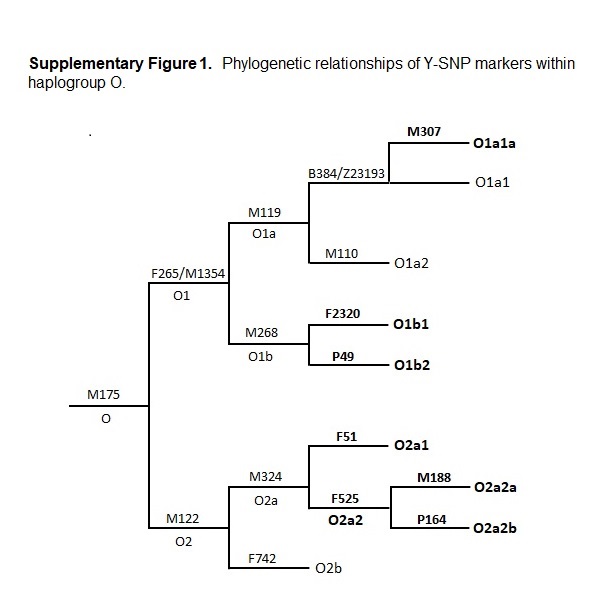

Supplement: Supplementary file 1 — Supplementary Figure 1. [file 41598_2024_52293_MOESM1_ESM.jpg]
