# Supplementary material for: Delineating the dispersal of Y-chromosome sub-haplogroup O2a2b-P164 among Austronesian-speaking populations
Source: Sci Rep. 2024 Jan 24;14:2066. doi: 10.1038/s41598-024-52293-z (PMC10808098; doi:10.1038/s41598-024-52293-z)
Supplement: Supplementary file 2 — Supplementary Table 1. [file 41598_2024_52293_MOESM2_ESM.docx]

Supplementary Table 1. List of populations with haplogroup O2a2b-P164 included in the study.

| Geographic Region  and Population | Population code | Language Family | Sample size | References |
| --- | --- | --- | --- | --- |
| **Oceania** |  |  |  |  |
| Kiritimati (Rep. Kiribati) | KIR | Austronesian | 114 | Palencia-Madrid et al., 2022 |
| West Samoa | WSM | Austronesian | 11 | Mirabal et al., 2012 |
| American Samoa (Manua) | ASM | Austronesian | 4 | Mirabal et al., 2012 |
| American Samoa (Tutuila) | ASM | Austronesian | 9 | Mirabal et al., 2012 |
| Tonga | TON | Austronesian | 25 | Mirabal et al., 2012 |
| Marquesas Is. | MRQ | Austronesian | 5 | Palencia-Madrid et al., 2019 |
| Leeward Society Is. | LWS | Austronesian | 7 | Hudjashov et al., 2018 |
| Maori | MAO | Austronesian | 4 | Hudjashov et al., 2018 |
| **South East Asia** |  |  |  |  |
| Taiwan – Han (Hakka) | TWH | Sino-Tibetan | 12 | Trejaut et al., 2014 |
| Taiwan – Han (Minnan) | TWH | Sino-Tibetan | 17 | Trejaut et al., 2014 |
| Taiwan – Han (Miscelaneous Han, Urban) | TWH | Sino-Tibetan | 32 | Trejaut et al., 2014 |
| Taiwan - Amis | AMI | Austronesian | 14 | Trejaut et al., 2014 |
| Taiwan - Paiwan | PAI | Austronesian | 1 | Trejaut et al., 2014 |
| Taiwan - Puyuma | PUY | Austronesian | 3 | Trejaut et al., 2014 |
| Taiwan - Saisiyat | SAI | Austronesian | 1 | Trejaut et al., 2014 |
| Taiwan - Taroko | TAR | Austronesian | 1 | Trejaut et al., 2014 |
| Taiwan - Kategalan | KAT | Austronesian | 5 | Trejaut et al., 2014 |
| Taiwan - Pazeh | PAZ | Austronesian | 6 | Trejaut et al., 2014 |
| Taiwan - Siraya (Hwalien) | SIR | Austronesian | 7 | Trejaut et al., 2014 |
| Taiwan - Siraya (Jiali) | SIR | Austronesian | 9 | Trejaut et al., 2014 |
| Taiwan - Siraya (Pingtung) | SIR | Austronesian | 8 | Trejaut et al., 2014 |
| Taiwan - Siraya (Kaohsiung) | SIR | Austronesian | 15 | Trejaut et al., 2014 |
| Taiwan - Siraya (Tainan-Coast) | SIR | Austronesian | 7 | Trejaut et al., 2014 |
| Taiwan - Siraya (Tainan01) | SIR | Austronesian | 10 | Trejaut et al., 2014 |
| Taiwan - Siraya (Tainan02) | ST2 | Austronesian | 3 | Trejaut et al., 2014 |
| Taiwan - Papura | PAP | Austronesian | 6 | Trejaut et al., 2014 |
| Taiwan - Yunlin | YUN | Austronesian | 10 | Trejaut et al., 2014 |
| East Indonesia (Maluku Ambon) | EIT | Austronesian | 4 | Trejaut et al., 2014 |
| East Indonesia (Nusa Tenggara) | EIT | Austronesian | 1 | Trejaut et al., 2014 |
| East Indonesia (Sulawesi) | EIT | Austronesian | 2 | Trejaut et al., 2014 |
| East Indonesia (Timor) | EIT | Austronesian | 1 | Trejaut et al., 2014 |
| West Indonesia (Undefined) | WIJ | Austronesian | 1 | Trejaut et al., 2014 |
| West Indonesia (Java) | WIJ | Austronesian | 5 | Trejaut et al., 2014 |
| West Indonesia (Kalimantan East) | WIJ | Austronesian | 1 | Trejaut et al., 2014 |
| West Indonesia (Kalimantan West) | WIJ | Austronesian | 6 | Trejaut et al., 2014 |
| West Indonesia (Sumatra) | WIJ | Austronesian | 5 | Trejaut et al., 2014 |
| Philippines - Batan (Ivatan) | BAT | Austronesian | 2 | Trejaut et al., 2014 |
| Philippines - North Luzon | NOL | Austronesian | 1 | Trejaut et al., 2014 |
| Philippines - South Luzon | SOL | Austronesian | 10 | Trejaut et al., 2014 |
| Philippines - Mindanao Island | MIN | Austronesian | 4 | Trejaut et al., 2014 |
| Philippines - Palawan | PLW | Austronesian | 1 | Trejaut et al., 2014 |
| Philippines - Visayan Island | VIS | Austronesian | 10 | Trejaut et al., 2014 |
| Philippines (Undefined) | PHI | Austronesian | 2 | Trejaut et al., 2014 |
| Thailand | THA | Tai-Kadai | 14 | Trejaut et al., 2014 |
| **China** |  |  |  |  |
| Han (Fujian) | CFI | Sino-Tibetan | 11 | Trejaut et al., 2014 |
|  |  |  |  |  |
|  |  |  |  |  |
